# Adaptation and validation of the patient assessment of chronic illness care in United States community pharmacies

**DOI:** 10.1186/s12913-022-07697-w

**Published:** 2022-03-17

**Authors:** Omolola A. Adeoye-Olatunde, Naomi Pratt, David D. Kim, Evan Schmidt, Margie E. Snyder

**Affiliations:** 1 Department of Pharmacy Practice, Purdue University College of Pharmacy, Indianapolis, USA; 2 Present Affiliation: Meijer Pharmacy, 17000 Mercantile Blvd, Noblesville, IN 46060 USA; 3 Purdue University College of Pharmacy, West Lafayette, USA; 4 Present Affiliation: Deaconess Health System, Evansville, USA; 5 Present Affiliation: Baptist Health Lexington, Lexington, USA

**Keywords:** Community pharmacy, Patient assessment of chronic illness care, Validity

## Abstract

**Background:**

Roles for United States (US)-based community pharmacists in caring for persons with chronic conditions have greatly expanded. The Patient Assessment of Chronic Illness Care (PACIC) was developed to assess patients’ perspectives of care received. However, successful application of this instrument in community pharmacies is uncertain. The objective of this study was to adapt the PACIC for use in community pharmacies (CP-PACIC), assess validity of the CP-PACIC and examine CP-PACIC scale score differences relative to patient characteristics.

**Methods:**

This cross-sectional study surveyed chronically ill adults in Indiana, US who receive care from pharmacists in community pharmacies. The modified CP-PACIC scale consisted of 20-items, which were rated on a 5-point Likert scale from 0 (none of the time) to 4 (always). The total possible score ranged from 0 – 80. An exploratory factor analysis (EFA) was conducted to assess performance and dimensionality. CP-PACIC validity, including face validity, construct validity (via exploratory factor analysis) and internal consistency were assessed. Relationships between patient characteristics and scale scores were evaluated using appropriate statistical tests.

**Results:**

Five hundred forty-six respondents’ data were analyzed. EFA revealed a 2-factor solution (termed advanced pharmacy chronic illness care (AP) and traditional pharmacy chronic illness care (TP) subscales) accounting for 64.7% of variance; all 20 items were retained. The total 20-item CP-PACIC scale had a Cronbach’s alpha (internal consistency) of 0.96; with a 12-item AP subscale Cronbach’s alpha of 0.97 and 8-item TP subscale Cronbach’s alpha of 0.89. Median total score was 12.0 [interquartile range = 27.3]. Median CP-PACIC sores varied across many respondent demographics (i.e., survey administration type, age, sex, education, health condition, number of pharmacy services received, community pharmacy type) such as whether respondents participated in one or more pharmacy service or not (29 vs. 10; *p* < .001).

**Conclusions:**

Unlike the original 5-subscale (patient activation, delivery system design, goal setting, problem solving, and follow-up/coordination) PACIC, analysis demonstrated a 2-factor (AP, TP) solution for the CP-PACIC scale with good internal consistency. As there are no standardized evaluation tools that exist, community pharmacies could find great value in using this CP-PACIC tool to benchmark performance and inform quality improvement strategies for patient care delivery.

**Supplementary Information:**

The online version contains supplementary material available at 10.1186/s12913-022-07697-w.

## Introduction

In the United States (US), it is estimated that approximately half of all adults has one chronic health condition, and a quarter have two or more chronic health conditions [1]. By 2030, an estimated 171 million Americans will have multiple chronic conditions [2]. Persons with chronic conditions are at higher risk of mortality and the US spends 86% of healthcare dollars on persons with at least one chronic condition [2].

Physicians, patients and policy makers have expressed concerns with the US healthcare system’s ability to adequately address the healthcare needs of persons with chronic illnesses [3]. Furthermore, 80% of health conditions require a prescription medication [4]. Community pharmacists offer a potential solution to this challenge and demand. In the US, community pharmacists dispense prescriptions (medications prescribed by an authorized provider) to patients and are considered the most accessible healthcare professional to the public. The community pharmacy (also known as retail pharmacy) structure typically includes licensed pharmacists and pharmacy technicians and is based in the community open to the public. Over the past two decades, roles for US-based community pharmacists in the provision of care for persons with chronic conditions have greatly expanded. Services such as vaccinations, medication therapy management (MTM), disease state management, and diabetes education programs are now routinely offered in the community pharmacy setting in addition to traditional dispensing [4]. Yet, despite the role that community pharmacists play in managing chronic conditions, there are no widely-recognized evaluation tools that exist for patients’ assessment of their care in the community pharmacy setting.

To categorize the critical aspects of the provision of care for persons with chronic illnesses, Wagner et al. created an evidence-based framework of six components known as the Chronic Care Model (CCM) [5]. As use of the CCM expanded, the Assessment of Chronic Illness Care (ACIC) was developed for health care teams to assess the extent to which they were implementing CCM elements into practice [6]. Subsequently, the Patient Assessment of Chronic Illness Care (PACIC) was developed as a method to assess the implementation of CCM components of care from the patient perspective to reduce potential bias of clinicians’ evaluations [7]. The PACIC is composed of five subscales: patient activation, delivery system design, goal setting, problem solving, and follow-up/coordination. The 20-item survey has been validated for internal consistency throughout various adaptations and certain populations, such as older patients [8], those with hypertension [9], diabetes and in a variety of languages [10]. However, due to the primary care clinic setting for which the PACIC was intended, successful application of this instrument in the community pharmacy setting is uncertain.

Therefore, there is a critical need to develop a measurement that evaluates patients’ perceptions of their chronic illness care in the community pharmacy setting. Thus, the objective of this study was to adapt the PACIC for use in community pharmacies (CP-PACIC), assess validity (i.e., face validity, construct validity, internal consistency) of the CP-PACIC and examine CP-PACIC scale score differences relative to patient characteristics.

## Methods

### Study design, setting and recruitment

This study was a cross-sectional evaluation of survey data collected from adults residing in Indiana, United States through two recruitment mechanisms. First, we utilized a community pharmacy practice-based research network (PBRN), Medication Safety Research Network of Indiana (Rx-SafeNet) to recruit study sites [11]. Pharmacies were recruited following usual network practices (e.g., emails, phone calls). Investigators/research assistants recruited respondents at pharmacy locations that volunteered to be a study site. Second, we utilized the Indiana Clinical and Translational Sciences Institute (CTSI) research volunteer registry to recruit respondents [12]. Persons in this registry have provided their health information for the purposes of being matched to appropriate research studies [12].

Individuals were eligible to participate in the study if they were at least 18 years of age, had at least one chronic medical condition, and had visited any community pharmacy at least two times in the past 6 months. For the purposes of this study, we categorized community pharmacy into 5 community pharmacy types: independently-owned, chain, grocery store-based, mass merchandiser, health system/hospital outpatient. In the US, independently-owned community pharmacies are privately held retail pharmacies not operated or owned by a publicly traded company, and according to the National Council for Prescription Drug Programs (NCPDP), has 1 to 3 pharmacy locations under common ownership [13]. Comparatively, chain community pharmacies are publicly traded and have 4 or more locations [13]. Lastly, grocery store-based, mass merchandiser, and health system/hospital community pharmacies are retail pharmacies affiliated with a grocery-store, mass merchandiser and health system/hospital respectively.

Data collection occurred from November 2017 – May 2019 in order to meet the study sample size goal of at least 200 respondents via each recruitment mechanism. A well-recognized sample size “rule of thumb” for factor analysis (i.e., an absolute N greater than 200 provides adequate statistical power for analysis) was followed in determining the minimal sample size [14]. All study data were collected anonymously, informed consent was obtained, eligibility screening was performed, and the protocol received exempt approval status by the Indiana University Institutional Review Board. Reporting is in accordance with the Strengthening the Reporting of Observational Studies in Epidemiology (STROBE) guidelines for reporting cross-sectional studies [15].

### Survey development and administration

The survey instrument consisted of 11 basic demographic questions and the 20 CP-PACIC survey items (Additional file 2). Basic demographic questions were included in order to characterize the study population. The 20-item survey was adapted from the previously published PACIC [7], to assess care patients received from community pharmacists. Based on expert opinion (face validity) of pharmacist research team members, minor wording changes were made to the PACIC to address the patient’s pharmacy care as opposed to their care from a physician while still aligning the survey with elements of the CCM. For example, “When I received care from my doctor…” was changed to “When I received care from my pharmacists…” Similar to the PACIC, respondents rated how often they experienced the content described in each item during the past 6 months. Each survey item was rated on a 5-point Likert scale from 0 (none of the time) to 4 (always) with a total possible score range of 0—80. As many patients fill prescriptions at multiple pharmacy locations the survey instrument asked respondents to consider all community pharmacies that they utilize.

Investigators/research assistants administered electronic surveys in-person to patients picking up a prescription at participating Rx-SafeNet pharmacies, and a web link was provided to participants from the CTSI volunteer registry using REDCap™ (Research Electronic Data Capture) [16]. REDCap™ is a secure, web-based application designed to support data capture for research studies [16]. In-person respondents also had the option of completing a paper-based survey instrument, in which data were subsequently input to REDCap™ by a study investigator or research assistant. As a compensation for their time, respondents had the option to participate in a voluntary drawing for one of six gift cards, valued at $25 each.

### Data analysis

Respondents’ data were excluded from analyses if 7 or more (≥ 35%) of the 20 CP-PACIC items were skipped. Missing data were managed using pairwise deletion. Descriptive statistics were computed to summarize respondent demographics and CP-PACIC scale scores and *P* values were reported for comparing across the two (in-person or online) survey administration types. For continuous demographic variables, appropriate measures of central tendency (mean [standard deviation] or median [interquartile range]) and group difference tests (t-test or Mann–Whitney U) were computed and performed based on normality of data. Data were considered to be normally distributed if less than 50% of items exhibited skewedness and/or kurtosis. Count and percent (n (%)) were calculated for categorical data. Chi-square group difference tests were performed for nominal data and Mann–Whitney U tests were performed for ordinal data.

Construct validity was assessed via exploratory factor analyses (EFA) using the *Principal Axis Factoring (PAF) with Promax* rotation factor extraction method (extraction criterion: Eigenvalue > 1). This method first conducts an orthogonal Varimax rotation and then allows correlations between the factors in an attempt to improve the fit to simple structure. Therefore, if the factors are in fact uncorrelated with one another, that will be revealed by a Promax rotation [17, 18]. Although commonly used, we did not use Principal Component Analysis method as it treats factors as if they are not related, leading to overestimation of factor loadings. According to Widaman et al. [19], PAF is “more accurate in reproducing population loadings” compared to Principal Components Analysis and thus, is the preferred method of extraction. If EFA results were similar across the two administrative types we reran the EFA for the full dataset.

Internal consistency was assessed using Cronbach’s alpha coefficient. Item-to-item and item-total correlations were performed to evaluate relationships between items and total CP-PACIC scale and the identified subscale scores. Spearman’s Correlation, Kruskal–Wallis, or Mann–Whitney U tests were conducted to assess differences among respondent characteristics and total median or mean (as appropriate) CP-PACIC scale scores. When median total CP-PACIC scale scores were significantly different, we performed post-hoc pairwise tests to further assess potential differences as appropriate. Significance values were adjusted by Bonferroni correction for multiple comparisons. All statistics were deemed significant at an a priori alpha of 0.05.

## Results

A total of 546 respondents’ data were included in analyses. Six pharmacies, which included four health system outpatient and two independent pharmacies, were recruited from the PBRN to conduct in-person survey administration. All in-person eligible respondents (*n* = 223) completed the minimum number of items to be included in analyses. The number of individuals who were approached or screened for eligibility in-person was not recorded, thus, a response rate is not reported. Of the 400 eligible online respondents, 323 completed the survey, yielding a response rate of 73.4%. Respondent demographics are reported and compared by survey administration type in Table 1. Generally, respondents were middle-aged (mean [SD], 52 [15.3]), non-Hispanic White (82.8%), female (74.6%), with at least some college (80.1%) and reported having high blood pressure (48.4%) and taking three or more medications (77%).

**Table 1:** Respondent demographics by in-person (*n* = 223) and online (*n* = 323) survey administration types and total study sample (*N* = 546)

Characteristic	n^a^	In-Person	n^b^	Online	N^c^	Total
Age (years), mean [SD]^d**^	218	58 (14.8)	323	49 (14.6)	540	52 (15.3)
Sex, n (%)^e**^	218		322		540	
Female	–––-	129 (59.2)	–––-	274 (85.1)	–––-	403 (74.6)
Ethnicity, n (%)^e**^	214		321		535	
Not Hispanic/Latino	–––-	188 (87.9)	–––-	309 (96.3)	–––-	497 (92.9)
Hispanic or Latino	–––-	4 (1.9)	–––-	5 (1.6)	–––-	9 (1.7)
Prefer not to answer	–––-	22 (10.3)	–––-	7 (2.2)	–––-	29 (5.4)
Race, n (%)^ef^	223		323		546	
White^*^	–––-	174 (78.0)	–––-	278 (86.1)	–––-	452 (82.8)
Black or African American^*^	–––-	35 (15.7)	–––-	30 (9.3)	–––-	65 (11.9)
Other^g^	–––-	8 (3.5)	–––-	7 (2.1)	–––-	15 (2.7)
Prefer not to answer	–––-	5 (2.2)	–––-	7 (2.2)	–––-	12 (2.2)
Highest level of schooling, n (%)^h***^	216		322		538	
At least some college	–––-	134 (62.0)	–––-	297 (92.2)	–––-	431 (80.1)
Tobacco use, n (%)^h^	217		322		539	
Never tried tobacco	–––-	92 (42.4)	–––-	131 (40.7)	–––-	233 (41.4)
Experimented with tobacco a few times in the past	–––-	31 (14.3)	–––-	59 (18.3)	–––-	90 (16.7)
Used to use tobacco but quit	–––-	64 (29.5)	–––-	82 (25.5)	–––-	146 (27.1)
Use tobacco less than once a day	–––-	4 (1.8)	–––-	6 (1.9)	–––-	10 (1.9)
Use tobacco once or more a day	–––-	26 (12.0)	–––-	44 (13.7)	–––-	70 (13.0)
Health conditions, n (%)^ef^	222		323		545	
Diabetes	–––-	52 (23.4)	–––-	67 (20.7)	–––-	119 (21.8)
Coronary artery disease/heart disease^**^	–––-	26 (11.7)	–––-	17 (5.3)	–––-	43 (7.9)
Chronic pain	–––-	51 (23.0)	–––-	92 (28.5)	–––-	143 (26.2)
Heart failure	–––-	6 (2.7)	–––-	6 (1.9)	–––-	12 (2.2)
COPD (bronchitis/emphysema)	–––-	10 (4.5)	–––-	8 (2.5)	–––-	18 (3.3)
Osteoporosis	–––-	20 (9.0)	–––-	33 (10.2)	–––-	53 (9.7)
High blood pressure^***^	–––-	129 (58.1)	–––-	135 (41.8)	–––-	264 (48.4)
Asthma^*^	–––-	29 (13.1)	–––-	63 (19.5)	–––-	92 (16.9)
High cholesterol	–––-	61 (27.5)	–––-	92 (28.5)	–––-	153 (28.1)
Arthritis	–––-	58 (26.1)	–––-	100 (31.0)	–––-	158 (29.0)
Kidney disease^**^	–––-	20 (9.0)	–––-	12 (3.7)	–––-	32 (5.9)
Depression^***^	–––-	49 (22.1)	–––-	130 (40.2)	–––-	179 (32.8)
Other^i***^	–––-	44 (19.8)	–––-	151 (46.7)	–––-	195 (35.8)
Pharmacy services received, n (%)^e^	215		317		532	
One or more service^j^	–––-	57 (26.5)	–––-	68 (21.5)	–––-	125 (23.5)
Not sure	–––-	6 (2.8)	–––-	13 (4.1)	–––-	19 (3.6)
None	–––-	152 (70.7)	–––-	236 (74.4)	–––-	388 (72.9)
Number of prescription medications, n (%)^h*^	217		323		540	
Less than three medications		39 (18.0)		85 (26.3)		124 (23.0)
Three or more medications	–––-	178 (82.0)	–––-	238 (73.7)	–––-	416 (77.0)
Frequency of pharmacy visits, n (%)^h^	215		321		536	
Less than once a month		45 (20.9)		56 (17.4)		101 (18.8)
At least once a month	–––-	170 (79.1)	–––-	265 (82.6)	–––-	435 (81.2)
Type of community pharmacy used for prescription medication(s), n (%)^e***^	217		322		539	
Independently-owned	–––-	50 (23.0)	–––-	14 (4.3)	–––-	64 (11.9)
Chain	–––-	42 (19.4)	–––-	170 (52.8)	–––-	212 (39.3)
Grocery store-based pharmacy	–––-	60 (27.6)	–––-	53 (16.5)	–––-	113 (21.0)
Mass merchandiser	–––-	15 (6.9)	–––-	55 (17.1)	–––-	70 (13.0)
Health system/hospital outpatient	–––-	50 (23.0)	–––-	30 (9.3)	–––-	80 (14.8)

*Abbreviations*: *COPD* Chronic obstructive pulmonary disease

^***^ *p* ≤ 0.001; ** *p* ≤ 0.01; * *p* < 0 .05

^a^number of non-missing in-person responses for each item

^b^number of non-missing online responses for each item

^c^total number of non-missing responses for each item

^d^t-test

^e^chi-square

^f^Select all that apply item, responses are not mutually exclusive and do not sum to 100%

^g^Other races included American Indian/Alaska Native, Asian, and Native Hawaiian or other Pacific Islander

^h^Mann-Whitney U

^i^The 10 most frequently reported “other” health conditions included anxiety, attention deficit disorder/attention deficit hyperactivity disorder, autoimmune disorders, cancer, epilepsy, fibromyalgia, gastrointestinal disorders, polycystic ovary syndrome, sleeping disorders, thyroid disorders

^j^The most frequently reported “other” pharmacy services received included immunizations, high blood pressure, diabetes education, and medication therapy management

Loading properties were similar for majority (*n* = 18, 90%) of CP-PACIC items among in-person (*n* = 223) and online (*n* = 323) respondent groups and resulted in a nearly identical (only loading values differed) two-factor solution [see Additional file 1] as the full data set, thus, we report results from the full data set (*N* = 546). The EFA revealed a 2-factor solution accounting for 64.7% of the variance, in which all 20 items were retained (Table 2). Factor 1, termed “advanced pharmacy chronic illness care (AP) subscale,” consisted of 12 items and Factor 2, termed “traditional pharmacy chronic illness care (TP) subscale,” consisted of the remaining 8 items (Table 2). As median CP-PACIC item scores differed between in-person and online respondent groups (described below), PAF analyses were conducted by respondent group (data not shown).

**Table 2:** Factor loadings of CP-PACIC items using Promax rotation (*N* = 546)

CP-PACIC items	**PAF** ^a^	
	F1^b^	F2^c^
1. Asked for my ideas when we discussed treatment/medicine options		0.59
2. Given choices about treatment/medicine to think about		0.51
3. Asked to talk about any problems with my medicines or their effects		0.75
4. Given written materials of things I should do to improve my health		0.53
5. Satisfied that my care was well organized		0.84
6. Informed how what I did to take care of my illness influenced my health condition(s)		0.58
7. Asked to talk about my goals in caring for my illness	0.64	
8. Helped to set specific goals to improve my eating or exercise	0.80	
9. Given a copy of my treatment/medicine plan		0.59
10. Encouraged to go to a specific group or class to help me cope with my chronic illness	0.97	
11. Asked questions, either directly or on a survey, about my health habits	0.83	
12. Sure that my pharmacists thought about my values and my traditions when they recommended treatments to me		0.54
13. Helped to make a treatment/medicine plan that I could do in my daily life	0.53	
14. Helped to plan ahead so I could take care of my illness even in hard times	0.53	
15. Asked how my chronic illness affects my life	0.77	
16. Contacted after a visit to see how things were going	0.77	
17. Encouraged to attend programs in the community that could help me	1.02	
18. Referred or encouraged to talk with a dietician, health educator, or counselor	0.94	
19. Told how my visits with other types of health care providers, like doctors and nurse practitioners, helped my treatment	0.90	
20. Asked how my visits with other health care providers were going	0.82	

*Abbreviations*: *CP-PACIC* Community Pharmacy-Patient Assessment of Chronic Illness Care, *PAF* Principal Axis Factoring

^a^Pattern matrix loading factors are reported, 2 factors extracted, variance explained was 64.7%

^b^Cronbach’s alpha for Factor 1 “advanced pharmacy chronic illness care subscale” 0.97

^c^Cronbach’s alpha for Factor 2 “traditional pharmacy chronic illness care subscale” 0.89

The total CP-PACIC scale, AP subscale, and TP subscale, had good internal consistencies with Cronbach’s alpha being 0.96, 0.97, and 0.89 respectively. For the total 20-item CP-PACIC scale, inter-item correlations ranged from 0.25 – 0.84 whereas item-total correlations ranged from 0.54 – 0.84. In regards to the 12-item AP subscale, inter-item correlations ranged from 0.62 – 0.84 whereas item-total correlations ranged from 0.77 –0.85. Lastly, the 8-item TP subscale inter-item correlations ranged from 0.38 – 0.78 whereas item-total correlations ranged from 0.56 – 0.76.

Median item scores for the total CP-PACIC scale, AP subscale, and TP subscale, were 0 [1.5], 0 [IQR 1.0], and 1.0 [IQR 2.5] respectively. Median total scores for the total CP-PACIC scale, AP subscale, and TP subscale, were 12.0 [27.3], 1.0 [IQR 13.0], and 11.0 [IQR 15.0] respectively. Median item scores by respondent group for each of the 20 CP-PACIC items are reported in Table 3. Numeric responses to all 20 CP-PACIC survey items in the in-person survey administration group were statistically significantly higher than the online survey administration group (*p* < 0.001) thus, indicating in-person respondents had a more positive assessment of their community pharmacy-based care (Table 3).

**Table 3:** Comparison of in person and online respondents’ responses to the 20-item CP-PACIC scale.^a^

	**In Person** (*n* = 223)							**Online** (*n* = 323)						
CP-PACIC item	**Median (IQR)**	**Mean Rank**	0 **None of the time** n (%)	1 **A little of the time** n (%)	2 **Some of the time** n (%)	3 **Most of the time** n (%)	4 **Always** n (%)	**Median (IQR)**	**Mean** **Rank**	0 **None of the time** n (%)	1 **A little of the time** n (%)	2 **Some of the time** n (%)	3 **Most of the time** n (%)	4 **Always** n (%)
1. Asked for my ideas when we discussed treatment/medicine options^***^	2.0 (3)	348.4	74 (33.2)	20 (9.0)	53 (23.8)	32 (14.3)	44 (19.7)	0 (0)	221.8	244 (75.5)	23 (7.1)	29 (9.0)	15 (4.6)	12 (3.7)
2. Given choices about treatment/medicine to think about^***^	2.0 (3)	342.4	81 (36.3)	17 (7.6)	45 (20.2)	41 (18.4)	39 (17.5)	0 (1)	223.9	231 (71.5)	40 (12.4)	32 (9.9)	12 (3.7)	8 (2.5)
3. Asked to talk about any problems with my medicines or their effects^***^	3.0 (3)	320.7	41 (18.4)	20 (9.0)	27 (12.1)	42 (18.8)	93 (41.7)	2.0 (3)	240.2	121 (37.5)	40 (12.4)	38 (11.8)	55 (17.0)	69 (21.4)
4. Given written materials of things I should do to improve my health^***^	3.0 (4)	332.8	58 (26.0)	20 (9.0)	29 (13.0)	34 (15.2)	82 (36.8)	0 (2)	230.9	183 (56.7)	32 (9.9)	32 (9.9)	29 (9.0)	47 (14.6)
5. Satisfied that my care was well organized^***^	4.0 (1)	332.2	13 (5.8)	10 (4.5)	21 (9.4)	56 (25.1)	123 (55.2)	3.0 (4)	232.3	51 (15.8)	30 (9.3)	66 (20.4)	96 (29.7)	80 (24.8)
6. Informed how what I did to take care of my illness influenced my health condition(s)^***^	2.0 (4)	353.3	58 (26.0)	30 (13.5)	26 (11.7)	43 (19.3)	66 (29.6)	0 (1)	217.4	217 (67.2)	37 (11.5)	30 (9.3)	25 (7.7)	14 (4.3)
7. Asked to talk about my goals in caring for my illness^***^	1.0 (3)	336.5	103 (46.2)	29 (13.0)	33 (14.8)	25 (11.2)	33 (14.8)	0 (0)	228.0	273 (84.5)	14 (4.3)	20 (6.2)	10 (3.1)	6 (1.9)
8. Helped to set specific goals to improve my eating or exercise^***^	1.0 (2)	334.0	109 (48.9)	25 (11.2)	41 (18.4)	20 (9.0)	28 (12.6)	0 (0)	228.5	278 (86.1)	19 (5.9)	14 (4.3)	9 (2.8)	3 (0.9)
9. Given a copy of my treatment/medicine plan^***^	4.0 (3)	333.7	45 (20.2)	14 (6.3)	23 (10.3)	27 (12.1)	114 (51.1)	0 (3)	230.6	172 (53.3)	18 (5.6)	24 (7.4)	31 (9.6)	78 (24.1)
10. Encouraged to go to a specific group or class to help me cope with my chronic illness^***^	0 (2)	324.7	137 (61.4)	16 (7.2)	22 (9.9)	17 (7.6)	31 (13.9)	0 (0)	238.2	298 (92.3)	11 (3.4)	5 (1.5)	4 (1.2)	5 (1.5)
11. Asked questions, either directly or on a survey, about my health habits^***^	0 (2)	325.5	117 (52.5)	26 (11.7)	26 (11.7)	21 (9.4)	33 (14.8)	0 (0)	235	274 (84.8)	17 (5.3)	14 (4.3)	9 (2.8)	9 (2.8)
12. Sure that my pharmacists thought about my values and my traditions when they recommended treatments to me^***^	2.0 (4)	345.4	77 (34.5)	15 (6.7)	27 (12.1)	33 (14.8)	71 (31.8)	0 (1)	223.9	232 (71.8)	20 (6.2)	38 (11.8)	16 (5.0)	17 (5.3)
13. Helped to make a treatment/medicine plan that I could do in my daily life^***^	2.0 (3)	338.1	89 (39.9)	19 (8.5)	31 (13.9)	33 (14.8)	51 (22.9)	0 (0)	224.3	248 (77.7)	24 (7.5)	23 (7.2)	10 (3.1)	14 (4.4)
14. Helped to plan ahead so I could take care of my illness even in hard times^***^	1.0 (3)	328.1	101 (45.3)	18 (8.1)	36 (16.1)	21 (9.4)	47 (21.1)	0 (0)	230.6	252 (78.0)	25 (7.7)	20 (6.5)	10 (3.1)	15 (4.6)
15. Asked how my chronic illness affects my life^***^	1.0 (3)	331.1	111 (49.8)	22 (9.9)	30 (13.5)	25 (11.2)	35 (15.7)	0 (0)	232.7	271 (83.9)	18 (5.6)	12 (3.7)	14 (4.3)	8 (2.5)
16. Contacted after a visit to see how things were going^***^	0 (3)	332.5	112 (50.2)	25 (11.2)	27 (12.1)	26 (11.7)	33 (14.8)	0 (0)	232.8	275 (85.1)	17 (5.3)	13 (4.0)	11 (3.4)	7 (2.2)
17. Encouraged to attend programs in the community that could help me^***^	0 (2)	320.3	137 (61.4)	21 (9.4)	24 (10.8)	16 (7.2)	25 (11.2)	0 (0)	238.6	293 (90.7)	15 (4.6)	7 (2.2)	5 (1.5)	3 (0.9)
18. Referred or encouraged to talk with a dietician, health educator, or counselor^***^	0 (2)	318.2	132 (59.2)	18 (8.1)	24 (10.8)	18 (8.1)	31 (13.9)	0 (0)	242.7	275 (85.1)	18 (5.6)	14 (4.3)	9 (2.8)	7 (2.2)
19. Told how my visits with other types of health care providers, like doctors and nurse practitioners, helped my treatment^***^	0 (3)	325.9	117 (52.5)	22 (9.9)	28 (12.6)	21 (9.4)	35 (15.7)	0 (0)	234.0	272 (84.2)	24 (7.4)	13 (4.0)	9 (2.8)	5 (1.5)
20. Asked how my visits with other health care providers were going^***^	0 (3)	323.9	117 (52.5)	21 (9.4)	28 (12.6)	21 (9.4)	36 (16.1)	0 (0)	234.2	269 (83.3)	19 (5.9)	13 (4.0)	14 (4.3)	8 (2.5)

*Abbreviations*: *CP-PACIC* Community Pharmacy-Patient Assessment of Chronic Illness Care, *IQR* interquartile range

^***^ *p* ≤ 0.001; ** *p* ≤ 0.01; * *p* < 0 .05

^a^ Group differences computed via Mann–Whitney U tests

Table 4 reports CP-PACIC findings by respondent characteristic. The in-person median CP-PACIC scale score was significantly higher than online median score (29 vs. 8 respectively; *p* < 0.001). Age exhibited a weak positive correlation (*r*_s_ = 0.102, *p* = 0.018). Male, less educated respondents had significantly higher scale scores than their counterparts (29 vs. 11; *p* < 0.001). Respondents who indicated they had coronary artery disease or heart disease, did not have arthritis and had depression had significantly higher median scale scores compared to their counterparts (*p* = 0.006, 0.004, and < 0.001, respectively). Respondents who indicated they participated in one more pharmacy service had significantly higher median scale scores compared to those who had not participated in a pharmacy service (29 vs. 10; *p* < 0.001). Median CP-PACIC scale scores varied by type of community pharmacy used. Respondents who reported using independent pharmacies had a significantly higher median scale score compared to those using grocery, chain, or mass merchandiser pharmacies (35 vs. 20, 8, 8; *p* = 0.041, < 0.001, < 0.001, respectively).

**Table 4:** Median total 20-item CP-PACIC scale scores by patient respondent characteristic

Characteristic	n	Median total CP-PACIC scale score (IQR)	*P*-value
Respondent group type^a^	**546**	**–––––––––**	< .001***
In-person	223	29 (36.0)	
Online	323	8 (12.0)	
Age (years)^b^	**541**	**–––––––––**	.018*
Sex^a^	**540**	**–––––––––**	< .001***
Male	137	29 (39.0)	
Female	403	11 (19.0)	
Ethnicity^a^	**506**	**–––––––––**	**.729**
Not Hispanic/Latino	497	12 (27)	
Hispanic or Latino	9	20 (26)	
Race^c,d^	**522**	**–––––––––**	**.243**
White	444	12 (25.8)	
Black or African American	65	16 (33.5)	
Other	13	23 (36.0)	
Education^a^	**538**	**–––––––––**	< .001***
No college	107	29 (38.0)	
At least some college	431	11 (22.0)	
Tobacco use status^a^	**539**	**–––––––––**	**.991**
Never or former user	459	12 (27.0)	
Current user	80	11 (32.5)	
Health conditions			
**Diabetes** ^a^	**544**	**–––––––––**	**.205**
No	425	12 (26.5)	
Yes	119	15 (32.0)	
**Coronary artery disease/heart disease** ^a^	**545**	**–––––––––**	.006**
No	502	12 (26.0)	
Yes	43	25 (39.0)	
**Chronic pain** ^a^	**544**	**–––––––––**	**.102**
No	401	13 (26.0)	
Yes	143	10 (29.0)	
**Heart failure** ^a^	**545**	**–––––––––**	**.146**
No	533	12 (27.0)	
Yes	12	40 (53.3)	
**COPD (bronchitis/emphysema)** ^a^	**545**	**–––––––––**	**.176**
No	527	12 (27.0)	
Yes	18	22.5 (34.5)	
**Osteoporosis** ^a^	**545**	**–––––––––**	**.213**
No	492	12 (30.0)	
Yes	53	12 (17.5)	
**High blood pressure** ^a^	**545**	**–––––––––**	**.125**
No	281	12 (23.5)	
Yes	264	14 (30.0)	
**Asthma** ^a^	**545**	**–––––––––**	**.944**
No	453	13 (27.0)	
Yes	92	11.5 (29.8)	
**High cholesterol** ^a^	**545**	**–––––––––**	**.638**
No	392	13 (25.0)	
Yes	153	11 (32.0)	
**Arthritis** ^a^	**545**	**–––––––––**	.004**
No	387	13 (31.0)	
Yes	158	10 (21.3)	
**Kidney disease** ^a^	**545**	**–––––––––**	**.254**
No	513	12 (27.0)	
Yes	32	17 (37.0)	
**Depression** ^a^	**545**	**–––––––––**	< .001***
No	366	15 (31.0)	
Yes	179	9 (18.0)	
**Other** ^a,e^	**545**	**–––––––––**	< .001***
No	350	16 (32.5)	
Yes	195	9 (18.0)	
Pharmacy services received^a,f^	**511**	**–––––––––**	< .001***
None	386	10 (20.0)	
One or more service	125	29 (39.0)	
Number of prescription medications^a^	**540**	**–––––––––**	**.669**
Less than three medications	124	11.5 (22.5)	
Three or more medications	416	12 (29.5)	
Frequency of pharmacy visits^a^	**536**	**–––––––––**	**.225**
Less than once a month	101	11 (17.5)	
At least once a month	435	13 (30.0)	
Type of community pharmacy used for prescription medication(s), n (%)^c,g^	**539**	**–––––––––**	< .001***
Independently-owned	64	35 (43.0)	
Chain	212	8 (16.0)	
Grocery store-based pharmacy	113	20 (28.5)	
Mass merchandiser	70	8 (15.0)	
Health system/hospital outpatient	80	18 (30.0)	

*Abbreviations*: *COPD* Chronic obstructive pulmonary disease, *CP-PACIC* Community Pharmacy-Patient Assessment of Chronic Illness Care, *IQR* interquartile range

^***^ *p* ≤ 0.001; ** *p* ≤ 0.01; * *p* < 0 .05

^a^Group differences computed via Mann–Whitney U tests

^b^Relationship between age and CP-PACIC scores was examined via Spearman’s Correlation tests, *r*_s_ = .102

^c^Group differences computed via Kruskal–Wallis tests, post-hoc pairwise tests performed when median total CP-PACIC scale scores were significantly different. Significance values were adjusted by Bonferroni correction for multiple comparisons

^d^Race categorized as follows: White (identified as White only), Black or African American (identified as Black or African American OR Black or African American and another race), Other (identified as American Indian, Native Hawaiian/Pacific Islander, or any other combination of races excluding Black)

^e^The 10 most frequently reported “other” health conditions included anxiety, attention deficit disorder/attention deficit hyperactivity disorder, autoimmune disorders, cancer, epilepsy, fibromyalgia, gastrointestinal disorders, polycystic ovary syndrome, sleeping disorders, thyroid disorders

^f^ “Not sure” responses were treated as missing data and were not included in analysis

^g^Chain vs. mass merchandiser *p* = 1.000, Chain vs. independent, grocery, or health system *p* < .001***, Mass merchandiser vs. grocery *p* = .012*, Mass merchandiser vs. health system *p* = .023*, Mass merchandiser vs. independent *p* < .001***, Grocery vs. health system *p* = 1.000, Grocery vs. independent *p* = .041*, Health system vs. independent *p* = .083

## Discussion

This is the first study to adapt and validate the PACIC in the US community pharmacy setting. This research provides several valuable contributions, including a brief tool (CP-PACIC) that could be useful in assessing community pharmacy patients’ perceptions of the care they receive, an assessment of the CP-PACIC scale properties and a detailed account of Indiana community pharmacy patients’ perceptions of their chronic illness care pre-COVID pandemic.

Our EFA of the 20-item CP-PACIC revealed a 2-factor solution, which differs from the proposed five factor PACIC [7]. Studies in different settings suggest one, two, three and four factor solutions [20–25]. These studies include a version administered to United Kingdom patients with chronic disease resulting in a one-factor solution, a version administered to Australian patients with chronic disease resulting in a two-factor solution, a Finnish version among patients with type 2 diabetes in the primary care setting demonstrating a three-factor solution, and a version administered to US patients with type 2 diabetes in the primary care setting resulting in a four-factor solution. This might be due to differences in how patients traditionally interact with healthcare teams in various settings. We termed these two factors as “traditional pharmacy chronic illness care (TP) subscale” comprising 8 items related to medication management and “advanced pharmacy chronic illness care (AP) subscale” comprising 12 items related to pharmacy care beyond medication management. As indicated by median total subscale scores, patients rated TP (11.0 [IQR 15.0]) higher than AP (1.0 [IQR 13.0]). This was expected, as the majority (70.4%, Table 1) of respondents indicated not participating in any pharmacy services and our findings suggest patients who participate in one or more pharmacy service have significantly higher CP-PACIC scores than those who do not participate (median score of 29 vs. 10, *p* < 0.001; Table 4).

Generally, community pharmacy patients had low CP-PACIC scores (median [IQR], 12.0 [27.3]). This is likely due to minimal incorporation of chronic care model components when dispensing medications in US community pharmacies. Future research should examine use of the CP-PACIC in evaluating chronic care pharmacy services such as medication therapy management (MTM), which is a service designed to help optimize medication use in Medicare Part D (drug coverage) beneficiaries with multiple chronic conditions. Specifically, baseline CP-PACIC scores could inform MTM service adjustments and then follow-up CP-PACIC scores can be assessed to determine the impact of service adjustments. Sub-group analyses can also determine whether any changes in scores vary across patients with specific chronic conditions, such as heart disease, which many MTM patients are diagnosed with and was associated with higher median scores in our analyses. Furthermore, US pharmacy education programs have recently started to incorporate the Pharmacists’ Patient Care Process as a standard of care, mirroring several components of the chronic care model [26]. It is important to note that this study took place prior to the COVID-19 pandemic, in which pharmacy services and public health efforts (e.g., point of care testing, vaccine, medication access services etc.) were often more recognized globally [27]. Future research should administer the CP-PACIC to evaluate community pharmacy patients’ perception of care by recent pharmacy graduates and post-COVID-19 pandemic.

This study was conducted in one mid-western state in the US limiting representation of findings to different states and countries. Moreover, our sample was more female and formally educated than the general Indiana population [28]. Additionally, in-person survey administration was only conducted at independent and health system community pharmacies, findings could have differed if in-person data collection occurred at chain pharmacies. Although widely recognized, a rule of thumb was followed rather than formal sample size calculation. Nevertheless, our approach was appropriate as we had acceptable factor loadings (≥ 0.50) and we did not perform a confirmatory analysis, which requires a larger sample size [14].

## Conclusions

Unlike the original 5-subscale PACIC measure, analysis demonstrated a 2-factor solution for the CP-PACIC scale with good internal consistency. Although CP-PACIC scores were generally low, several patient characteristics, such as whether they’ve participated in pharmacy services, exhibited higher CP-PACIC scores. As there are no standardized evaluation tools that exist, community pharmacies could find great value in using this CP-PACIC tool to benchmark performance and inform quality improvement strategies for patient care delivery.

## Supplementary Information

**Additional file 1.**

**Additional file 2.**

## Data Availability

The datasets used and/or analyzed during the current study are available from the corresponding author on reasonable request.

## Abbreviations

AP: Advanced pharmacy chronic illness care
ACIC: Assessment of Chronic Illness Care
CCM: Chronic Care Model
CP-PACIC: Community Pharmacy—Patient Assessment of Chronic Illness Care
CTSI: Clinical and Translational Sciences Institute
EFA: Exploratory factor analyses
MTM: Medication therapy management
Rx-SafeNet: Medication Safety Research Network of Indiana
PACIC: Patient Assessment of Chronic Illness Care
PBRN: Pharmacy practice-based research network
PAF: Principal Axis Factoring
REDCap™: Research Electronic Data Capture
STROBE: Strengthening the Reporting of Observational Studies in Epidemiology
TP: Traditional pharmacy chronic illness care
US: United States
