# Supplementary material for: Adaptation and validation of the patient assessment of chronic illness care in United States community pharmacies
Source: BMC Health Serv Res. 2022 Mar 17;22:355. doi: 10.1186/s12913-022-07697-w (PMC8929461; doi:10.1186/s12913-022-07697-w)
Supplement: Supplementary file 1 — Additional file 1. [file 12913_2022_7697_MOESM1_ESM.docx]

**Additional File 1** Factor loadings of the CP-PACIC items for in-person and online survey administration.

**Table 1.** Factor loadings of CP-PACIC items for in-person survey administration using Promax rotation (N = 223).

| CP-PACIC items | Principal Axis Factoring  Factor | |
| --- | --- | --- |
|  | 1 | 2 |
| 1. Asked for my ideas when we discussed treatment/medicine options |  | .707 |
| 2. Given choices about treatment/medicine to think about |  | .593 |
| 3. Asked to talk about any problems with my medicines or their effects |  | .743 |
| 4. Given written materials of things I should do to improve my health |  | .399 |
| 5. Satisfied that my care was well organized |  | .790 |
| 6. Informed how what I did to take care of my illness influenced my health condition(s) |  | .623 |
| 7. Asked to talk about my goals in caring for my illness | .547 |  |
| 8. Helped to set specific goals to improve my eating or exercise | .760 |  |
| 9. Given a copy of my treatment/medicine plan |  | .509 |
| 10. Encouraged to go to a specific group or class to help me cope with my chronic illness | .925 |  |
| 11. Asked questions, either directly or on a survey, about my health habits | .803 |  |
| 12. Sure that my pharmacists thought about my values and my traditions when they recommended treatments to me |  | .610 |
| 13. Helped to make a treatment/medicine plan that I could do in my daily life | .477 |  |
| 14. Helped to plan ahead so I could take care of my illness even in hard times | .512 |  |
| 15. Asked how my chronic illness affects my life | .706 |  |
| 16. Contacted after a visit to see how things were going | .823 |  |
| 17. Encouraged to attend programs in the community that could help me | .966 |  |
| 18. Referred or encouraged to talk with a dietician, health educator, or counselor | .970 |  |
| 19. Told how my visits with other types of health care providers, like doctors and nurse practitioners, helped my treatment | .884 |  |
| 20. Asked how my visits with other health care providers were going | .812 |  |

**Table 2.** Factor loadings of CP-PACIC items for online survey administration using Promax rotation (N = 323).

|  |  | Principal Axis Factoring | | |
| --- | --- | --- | --- | --- |
| CP-PACIC items | | | Factor | |
|  |  |  | 1 | 2 |
| 1. Asked for my ideas when we discussed treatment/medicine options | | |  | .428 |
| 2. Given choices about treatment/medicine to think about | | | .423 |  |
| 3. Asked to talk about any problems with my medicines or their effects | | |  | .725 |
| 4. Given written materials of things I should do to improve my health | | |  | .559 |
| 5. Satisfied that my care was well organized | | |  | .739 |
| 6. Informed how what I did to take care of my illness influenced my health condition(s) | | |  | .470 |
| 7. Asked to talk about my goals in caring for my illness | | | .736 |  |
| 8. Helped to set specific goals to improve my eating or exercise | | | .819 |  |
| 9. Given a copy of my treatment/medicine plan | | |  | .520 |
| 10. Encouraged to go to a specific group or class to help me cope with my chronic illness | | | .970 |  |
| 11. Asked questions, either directly or on a survey, about my health habits | | | .814 |  |
| 12. Sure that my pharmacists thought about my values and my traditions when they recommended treatments to me | | |  | .438 |
| 13. Helped to make a treatment/medicine plan that I could do in my daily life | | | .513 |  |
| 14. Helped to plan ahead so I could take care of my illness even in hard times | | | .469 | .306 |
| 15. Asked how my chronic illness affects my life | | | .779 |  |
| 16. Contacted after a visit to see how things were going | | | .592 |  |
| 17. Encouraged to attend programs in the community that could help me | | | 1.040 |  |
| 18. Referred or encouraged to talk with a dietician, health educator, or counselor | | | .836 |  |
| 19. Told how my visits with other types of health care providers, like doctors and nurse practitioners, helped my treatment | | | .839 |  |
| 20. Asked how my visits with other health care providers were going | | | .743 |  |
