# Supplementary material for: Adaptation and validation of the patient assessment of chronic illness care in United States community pharmacies
Source: BMC Health Serv Res. 2022 Mar 17;22:355. doi: 10.1186/s12913-022-07697-w (PMC8929461; doi:10.1186/s12913-022-07697-w)
Supplement: Supplementary file 2 — Additional file 2. [file 12913_2022_7697_MOESM2_ESM.docx]

**CP-PACIC**

Staying healthy can be difficult when you have a chronic illness. We would like to learn about the type of help with your condition you get from your pharmacists. Your answers will be kept confidential and will not be shared with anyone else.

**Over the past 6 months, when I received care from my pharmacists, I was:**

|  | None of the time | A little of the time | Some of the time | Most of the time | Always |
| --- | --- | --- | --- | --- | --- |
| 1. Asked for my ideas when we discussed treatment/medicine options |  |  |  |  |  |
| 2. Given choices about treatment/medicine to think about |  |  |  |  |  |
| 3. Asked to talk about any problems with my medicines or their effects |  |  |  |  |  |
| 4. Given written materials of things I should do to improve my health |  |  |  |  |  |
| 5. Satisfied that my care was well organized |  |  |  |  |  |
| 6. Informed how what I did to take care of my illness influenced my health condition(s) |  |  |  |  |  |
| 7. Asked to talk about my goals in caring for my illness |  |  |  |  |  |
| 8. Helped to set specific goals to improve my eating or exercise |  |  |  |  |  |
| 9. Given a copy of my treatment/medicine plan |  |  |  |  |  |
| 10. Encouraged to go to a specific group or class to help me cope with my chronic illness |  |  |  |  |  |
| 11. Asked questions, either directly or on a survey, about my health habits |  |  |  |  |  |
| 12. Sure that my pharmacists thought about my values and my traditions when they recommended treatments to me |  |  |  |  |  |
| 13. Helped to make a treatment/medicine plan that I could do in my daily life |  |  |  |  |  |
| 14. Helped to plan ahead so I could take care of my illness even in hard times |  |  |  |  |  |
| 15. Asked how my chronic illness affects my life |  |  |  |  |  |
| 16. Contacted after a visit to see how things were going |  |  |  |  |  |
| 17. Encouraged to attend programs in the community that could help me |  |  |  |  |  |
| 18. Referred or encouraged to talk with a dietician, health educator, or counselor |  |  |  |  |  |
| 19. Told how my visits with other types of health care providers, like doctors and nurse practitioners, helped my treatment |  |  |  |  |  |
| 20. Asked how my visits with other health care providers were going |  |  |  |  |  |

**What is your age?**

________________________________________________________________

**What is your sex?**

- Male
- Female
- Prefer not to answer

**What is your ethnicity?**

- Non-Hispanic or Latino
- Hispanic or Latino
- Prefer not to answer

**What is your race?**

- American Indian/Alaska native
- Black or African American
- White
- Asian
- Native Hawaiian or other pacific islander
- Prefer not to answer

**What is your highest degree or level of school you have completed? (If currently enrolled, mark the previous grade or highest degree received)**

- No schooling completed
- Nursery to 11th grade
- 12th grade (no degree)
- High School Diploma
- GED or alternative credential
- Some college
- Associate's degree
- Bachelor's degree
- Advanced degree
- Prefer not to answer

**Which of the following best describes your tobacco use (cigarettes, cigars, pipes, snuff, chew, or hookah)?**

- Use tobacco once or more a day
- Use tobacco less than once a day
- Used to use tobacco but quit
- Experimented with tobacco a few times in the past
- Never tried tobacco

**Which of the following health conditions do you have?**

- Diabetes
- Coronary artery disease/Heart disease
- Chronic pain
- Heart failure
- Chronic pulmonary obstructive disease (bronchitis/emphysema)
- Osteoporosis
- High blood pressure
- Asthma
- High cholesterol
- Arthritis
- Kidney disease
- Depression
- Other: ________________________________________________

**Which of the following services have you participated in at your pharmacy? (Check all that apply)**

- Smoking cessation
- Weight loss
- Medication therapy management
- Asthma management
- High blood pressure management
- High cholesterol management
- Diabetes education
- Medication synchronization (MedSync)
- Other: ________________________________________________
- Not sure
- None

**How often do you typically visit your usual pharmacy?**

- More than once a week
- About once a week
- More than once a month
- About once a month
- About once every three months
- Less than once every three months

**About how many different prescription medications do you get filled at your usual pharmacy?**

- 0
- 1-2
- 3-4
- 5-6
- 7-8
- 9-10
- 11-12
- 13-14
- 15+

**From which type of pharmacy do you get most of your prescription medications?**

- Retail/Community independently-owned pharmacy (i.e. your pharmacy has 4 stores/locations or less)
- Retail/Community chain pharmacy (i.e. 4 or more stores; e.g. CVS, Walgreens)
- Retail/Community grocery store-based pharmacy (e.g. Kroger)
- Retail/Community mass merchandiser (e.g. Walmart, Meijer, CVS inside of Target, Costco, Sam's Club)
- Health System/Hospital Outpatient pharmacy (e.g. Eskenazi)

End of Block: Default Question Block

Survey ID:_________________
